# Antioxidant activity and peroxidase inhibition of Amazonian plants extracts traditionally used as anti-inflammatory

**DOI:** 10.1186/s12906-016-1061-9

**Published:** 2016-02-27

**Authors:** Fabiano S. de Vargas, Patricia D. O. Almeida, Ana Paula A. de Boleti, Maria M. Pereira, Tatiane P. de Souza, Marne C. de Vasconcellos, Cecilia Veronica Nunez, Adrian M. Pohlit, Emerson S. Lima

**Affiliations:** Faculdade de Ciências Farmacêuticas, Universidade Federal do Amazonas - UFAM, Rua Alexandre Amorim, 330, Bairro Aparecida, Cep: 69010-300, Manaus, Amazonas Brazil; Coordenação de Tecnologia e Inovação-CoTI, Instituto Nacional de Pesquisas da Amazônia-INPA, Avenida André Araújo, Petrópolis, CEP: 69067375, Manaus, Amazonas Brazil

**Keywords:** Amazon medicinal plants, Antioxidants, Citotoxicity, Reactive oxygen species, Free radical scavenging

## Abstract

**Background:**

The Amazon is the largest rainforest in the world and is home to a rich biodiversity of medicinal plants. Several of these plants are used by the local population for the treatment of diseases, many of those with probable anti-inflammatory effect. The aim of the present investigation was to evaluate the in vitro antioxidant and anti-peroxidases potential of the ethanol extracts of five plants from the Brazilian Amazon (*Byrsonima japurensis*, *Calycophyllum spruceanum*, *Maytenus guyanensis*, *Passiflora nitida* and *Ptychopetalum olacoides*).

**Methods:**

DPPH, ABTS, superoxide anion radical, singlet oxygen and the β-carotene bleaching methods were employed for characterization of free radical scavenging activity. Also, total polyphenols were determined. Antioxidant activities were evaluated using murine fibroblast NIH3T3 cell. Inhibition of HRP and MPO were evaluated using amplex red® as susbtract.

**Results:**

The stem bark extracts of *C. spruceanum* and *M. guyanensis* provided the highest free radical scavenging activities. *C. spruceanum* exhibited IC_50_ = 7.5 ± 0.9, 5.0 ± 0.1, 18.2 ± 3.0 and 92.4 ± 24.8 μg/mL for DPPH^•^, ABTS^+•^, O_2_^-•^ and ^1^O_2_ assays, respectively. *P. olacoides* and *C. spruceanum* extracts also inhibited free radicals formation in the cell-based assay. At a concentration of 100 μg/mL, the extracts of *C. spruceanum, B. japurensis* inhibited horseradish peroxidase by 62 and 50 %, respectively. *C. spruceanum, M. guyanensis, B. japurensis* also inhibited myeloperoxidase in 72, 67 and 56 %, respectively.

**Conclusions:**

This work supports the folk use these species that inhibited peroxidases and exhibited significant free radical scavenging and antioxidant activities what can be related to treatment of inflammation.

## Background

The enormous biodiversity of the Amazon jungle has potential as the source of new natural products. Many species that are still essentially unknown to science have been used for centuries by local populations for treating a variety of illnesses [1]. The pursuit of new therapeutic alternatives and the development of new drugs starting from natural products have been the underlying motives for chemical and pharmacological studies. Regions having abundant flora, medicinal plants and a rich traditional knowledge, as is the case of the Amazon forest, are especially attractive given the relative low number of publications on plants from this part of the world [2].

Plants used in traditional medicine can provide diverse secondary metabolites with antioxidant potential most of which are phenolic compounds [3, 4] such as flavonoids and tannins. Flavonoids are of particular interest because of their antioxidant activity and ability to act as scavengers of oxygen radicals. The antioxidant capacity of these phenolic compounds is mainly due to their redox properties, which allow them to act as reducing agents, hydrogen donors and singlet oxygen quenchers, or decomposing peroxides [3, 5].

Reactive oxygen species (ROS) such as singlet oxygen (^1^O_2_), super oxide anion (O_2_^-^) hydroxyl radical (∙OH) and hydrogen peroxide (H_2_O_2_) are often generated as by-products of biological reactions or from exogenous factors. These reactive species cause oxidative damage in reactions with nearly every molecule found in living cells, including DNA [6]. Thus, excess ROS must be eliminated by an antioxidant system. They play important roles in aging and in the pathogenesis of age related disorders such as cancer, hypertension, atherogenesis, Alzheimer’s disease and Parkinson’s disease [7].

In order to gain further knowledge of folk uses and traditional plants in the Amazon, the purpose of this research is to evaluate in vitro antioxidant and peroxidasic activity of ethanolic extract of the leaves and bark of *Byrsonima japurensis* A. Juss.*, Calycophyllum spruceanum* (Benth.) Hook. f. ex K. Schum.*, Maytenus guyanensis* Klotzch.*, Passiflora nitida* Kunth. and *Ptychopetalum olacoides* Benth.

## Methods

### Chemicals

Ultrapure water was prepared using a Millipore Direct® Q_3_ (Millipore Corp., MA, U.S.A.) and was used throughout. All remaining reagents were of the highest purity available and obtained from the Sigma Chemical Company (St. Louis, MO, U.S.A.).

### Plant materials

Plant samples were collected in different locations in the State of Amazonas, Brazil. *Calycophyllum spruceanum* (Benth.) Hook. f. ex K. Schum. (common name mulateiro), *Byrsonima japurensis* A. Juss. (common name saratudo), *Passiflora nítida* Kunth. (common name maracujá-do-mato) were collected at Lago do Purupuru (Purupuru Lake) in Careiro Castanho municipality. *Maytenus guyanensis* Klotzch. (common name chichuá) and *Ptychopetalum olacoides* Benth. (common name muirapuama,) were collected in the region near the city of Benjamin Constant (Table 1). Voucher samples were deposited at the National Institute for Amazon Research (INPA) and Amazonas Federal University (UFAM) Herbariums. Plants were identified by MSc. Carlos Alberto Cid Ferreira, MSc. Carlos Henrique Franciscon and staff from INPA’s Botany Department (CPBO). *Byrsonima japurensis* A. Juss. (voucher number - 127281) - Malpighiaceae family; *Calycophyllum spruceanum* (Benth.) Hook. f. ex K. Schum. (174714) - family; *Maytenus guyanensis* Klotzch. (157502) - Celastraceae family; *Passiflora nitida* Kunth. (209547) - Passifloraceae family; and *Ptychopetalum olacoides* Benth. (138278) - Olacaceae family.

**Table 1:** Medicinal plants used in this study

Plant species	Common name	Part used	Traditional use
*B. japurensis*	Saratudo	Stem bark	Indicated against various inflammatory disorders, especially in the uterus and prostate
*C. spruceanum*	Mulateiro	Stem bark	For age spots, cuts, diabetes, eye infections, ovarian problems, scars, scrapes, skin fungi, skin parasites, skin problems, wrinkles, and wounds, and as an antioxidant and cosmetic
*M. guyanensis*	Chichuá	Stem bark	Used as a stimulant, tonic and muscle relaxant, to relieve arthritis, rheumatism, hemorrhoids, swollen kidney, skin rashes, skin cancer prevention.
*P. nitida*	Maracujá-do-mato	Leaves	Treatment of gastrointestinal disorders
*P. olacoides*	Muirapuama	Leaves	Used as tonic neuromuscular, neurasthenia, impotence, menstrual disorders, dysentery. It also has stimulant properties of the central nervous system.

### Preparation of extracts

Leaves and bark of *M. guyanensis* and *P. olacoides* were dried in the shade at room temperature only. *C. spruceanum*, *B. japurensis* and *P. nitida* were first dried in the shade and then further dried in an oven with circulation of air at 45 °C for 48 h. Dried plant samples were ground and stored in sealed bags until extraction was performed. Ground *C. spruceanum*, *B. japurensis* and *P. nitida* were extracted with ethanol for 20 min in an ultrasound bath then macerated in the same solvent for 72 h. This extraction procedure was repeated 2× and the extracts were combined. Ground *P. olacoides* was macerated in ethanol (2 × 1 week) and the extracts were combined and *M. guyanensis* was extracted with ethanol in a soxhlet apparatus (1 × 8 h). Each solution or extract was filtered and the solvents removed on a rotary evaporator under reduced pressure and low bath temperature (50 °C), then freeze-dried to obtain each dried extract which was then used in the antioxidant tests. The extraction procedures were performed and afforded yield and other data as presented in Table 2 [8, 9].

**Table 2:** Experimental procedures used in preparation of extracts

Species	Part used	Solvent	Oven drying	Extraction	Time	Solvents evaporation
*B. japurensis* ^1^	Bark	Ethanol	Room temperature	Cold Maceration	48 h	Evaporator rotative and lyophilization
*C.spruceanum* ^1^	Bark	Ethanol	Room temperature	Cold Maceration	48 h	Evaporator rotative and lyophilization
*M. guyanensis* ^2^	Bark	Ethanol	Hot air (45 °C/48 h)	Cold Maceration with ultrasound bath	20 min	Evaporator rotative and lyophilization
*P. nitida* ^1^	Leaf	Ethanol	Room temperature	Cold Maceration	48 h	Evaporator rotative and lyophilization
*P. olacoides* ^1^	Leaf	Ethanol	Hot air (45 °C/48 h)	Cold Maceration with ultrasound bath	20 min	Evaporator rotative and lyophilization

1 – Simões, et al. 2004 [9]. 2 – Macari, et al. 2006 [8]

### Determination of total polyphenols

The test was performed using the Folin-Ciocalteu colorimetric method as described by Singleton and Rossi [10]. Each dried plant extract was dissolved in EtOH at a concentration of 10 mg/mL. Each test solution was transferred to a test tube. Then, distilled H_2_O (400 μL) and Folin-Ciocalteau reagent (160 μL) were added. After homogenization in a vortex apparatus, 10.6 % aqueous Na_2_CO_3_ (4 mL) was added. After incubation for 3 min, the absorbance was measured at 715 nm in a spectrophotometer (Ultrospec 2000 UV/Vis, Pharmacia Biotech, England). The total polyphenol content was expressed in milligrams (mg) of EGA (equivalents of gallic acid) per gram (g) of extract. All analyses were performed in triplicate.

### DPPH assay

This assay was performed as described by [11]. Initially, a DPPH (diphenylpicrylhydrazyl) solution (0.8 mmol/L) was prepared in MeOH. The final volume of each test well (350 μL) was made up of extract dissolved in MeOH (250 μL), or pure MeOH in the case of controls, and 0.8 mM DPPH solution (100 μL). An initial spectrophotometric reading of each extract in MeOH was performed (blank, Abs_1_). After addition of DPPH solution to wells, the test plate was allowed to stand in the dark at room temperature for 30 min and then the absorbance was measured at 517 nm (Abs_2_) using a test plate reader (TP-Reader, Thermoplate, Italy). TROLOX® was used as antioxidant standard. The results were obtained using the following formula:

$$ \%\  Inhibition = 100 \times \left[ 1\ \mathit{\hbox{--}}\ \left( Ab{s}_2 sample\ \mathit{\hbox{--}}\  Ab{s}_1 sample\right)/ Ab s\  control\right] $$

### ABTS assay

This method is based on the oxidation of ABTS [2,2′-azino-bis(3-ethylbenzthiazoline-6-sulphonate)] as described by Re et al. [12]. An oxidized ABTS (ABTS^+^) solution was prepared by adding ABTS (10 mg) in H_2_O (5 mL) to 5 mM K_2_S_2_O_8_ (10 mL). The resulting solution was allowed to stand 24 h prior to use. The assay was performed in 96-well test plates. Each well was charged with ABTS^+^ solution (40 μL), deionized H_2_O (60 μL) and a solution (250 μL) of plant extract (0.1–10 g/L) in deionized H_2_O or pure deionized H_2_O (250 μL) for control wells. First an absorbance reading was performed on each well containing only extract dissolved in deionized H_2_O as a blank (Abs_1_). After addition of ABTS^+^ solution, the plate was allowed to stand at room temperature and ambient light for 15 min, then a final reading (Abs_2_) was performed at 714 nm on a test plate reader (TP-Reader, Thermoplate, Italy). Trolox® was used as antioxidant standard. Antioxidant activity of extracts was calculated using the following formula:

$$ \%\  Inhibition = 100\left[ 1\ \mathit{\hbox{--}}\left( Ab{s}_2\kern0.1em  sample\ \mathit{\hbox{--}}\  Ab{s}_1\kern0.1em  sample\right)/ Ab s\  control\right] $$

### Superoxide anion radical assay

The method used was described by Ozturk et al. [13]. Test solutions were prepared by dissolving each dry extract in EtOH (10 mg/mL), and 16 mM Tris–HCl pH 8.0 buffer was used as solvent. The wells of a test plate were charged with extract sample (50 μL), 250 μM nitrobluetetrazolium (NBT, 100 μL) and 390 μM NADH (100 μL). Absorbance was registered on a test plate reader (TP-Reader, Thermoplate, Italy) at 560 nm (Abs_1_). Then, 10 μM phenazine methylsulfate (PMS, 100 μL) was added and the plate was left for 5 min at 25 °C. Next, absorbance was again registered (Abs_2_). Gallic acid was used as antioxidant reference standard. Inhibition was calculated using the following formula:

$$ \%\  Inhibition = 100\left[ 1\ \mathit{\hbox{--}}\left( Ab{s}_2\kern0.5em  sample\ \mathit{\hbox{--}}\  Ab{s}_1\kern0.5em  sample\right)/ Ab s\  control\right] $$

### Singlet oxygen assay

This test was performed in accordance with the method described by Gregianini et al. [14]. Briefly, a 0.8 mmol/L rubrene solution was prepared in CHCl_3_ and protected from incident light. Sample solutions (100–5000 μg/mL) of each extract were prepared in CHCl_3_. The test was performed in a clear glass test tube to which was added rubrene solution (2 mL) and extract test solution (20 μL). Next, test tubes were kept at a distance of 20 cm from a 40 W fluorescent lamp for 120 min at room temperature. DABCO (1,4-diazabicyclo[2.2.2]octane) was used as antioxidant standard. The absorbance of the test solutions were measured at 440 nm on an spectrophotometer (Ultrospec 2000 UV/Vis - Pharmacia Biotech, Cambridge, England) before (Abs_1_) and after the period of exposure to fluorescent light (Abs_2_). All tests were performed in triplicate. Inhibition was calculated using the following formula:

$$ \%\  Inhibition = 100\left[ 1\ \mathit{\hbox{--}}\ \left( Ab{s}_2\kern0.2em  sample\ \mathit{\hbox{--}}\ {A}_1\kern0.2em  sample\right)/\left( Ab{s}_2\kern0.2em  control\mathit{\hbox{--}}\  Ab{s}_1\kern0.2em  control\right)\right] $$

### β -carotene bleaching assay

The antioxidant activity of extracts was evaluated using the β-carotene-linoleic acid method described by Miller [15]. The samples were diluted in EtOH: H_2_O (1:1) to a concentration of 100 μg/mL. 10 μL of extract solutions, water (blank) or BHT (butylated hydroxytoluene, standard) at the same concentraction of the extracts were added to the wells of a 96-well plate. Next, β-carotene (2.0 mg) was dissolved in CHCl_3_ (1.0 mL). A portion of the resulting solution (150 μL) was added to linoleic acid (50 μL), Tween 80 emulsifier mixture (200 μL) and CHCl_3_ (500 μL) in an Erlenmeyer flask and homogenized. After evaporation of the CHCl_3_ under a flow of N_2_, distilled H_2_O (25 mL) previously saturated with air was added followed by vigorous shaking for 30 min. A portion of the resulting emulsion (240 μL) was transferred to each well and the zero time absorbance was immediately measured at 470 nm using an ELISA reader Multimode Detector DTX-800 microplate reader (Beckman Coulter, CA, USA). The emulsion system was incubated for 2 h at 50 °C and the absorbance was measured every 15 min.

### Antioxidant activity in cell

This assay was performed as described by Wolfe & Liu [16]. NIH3T3 cells (fibroblast murine) were seeded at 6 × 10^4^/well on a 96-well plate in 100 μL of growth medium (DMEM) and incubated for 24 h at 37 °C. 24 h after seeding, the medium was removed and the cells were washed with PBS. Triplicate wells were treated for 1 h with 100 μL medium with extract at a concentration of 20 μg/mL and 25 μM DCFH-DA. After the treatment period, the cells were washed with PBS (100 μL). Then 600 μM ABAP was applied to the cells in HBSS (100 μL), and the fluorescence was measured in ELISA reader Multimode Detector DTX-800 microplate reader (Beckman Coulter, CA, USA) with emission at 538 nm and excitation at 485 nm every 5 min for 1 h. Quercetin in DMSO (20 μg/mL) and saline solution were used as positive and negative controls, respectively. Each plate included triplicate control and blank wells: control wells contained cells treated with DCFH-DA and oxidant; negative control wells contained cells treated with dye and PBS without oxidant.

### Peroxidase inhibition activities

In the presence of horseradish peroxidase (HRP) or myeloperoxidase (MPO) Amplex Red® reagent (10-acetyl-3,7-dihydroxyphenoxazine) reacts with H_2_O_2_ in to produce highly fluorescent resorufin which can be used as a highly sensitive probe for the presence of H_2_O_2_ [17]. Each extract was diluted in DMSO to a concentration of 100 μg/mL, In each well of a 96-well flat bottom microplate extract solution (10 μL), 10 nM peroxidase enzyme (HRP or MPO) solution (5 μL), 50 μM Amplex Red® reagent (5 μL), and 50 mM pH 7.4 phosphate buffer solution (170 μL) were added. After incubation for 5 min at r.t., 50 μM H_2_O_2_ (10 μL) was added using a multichannel pippete. The kinetic reaction was monitored spectrophotometrically in an ELISA reader Multimode Detector DTX-800 microplate reader (Beckman Coulter, CA, USA) every 30 s for 15 min. (λ = 550 nm). All the extracts were treated similarly, and the percent inhibition was calculated according to the following equation:

$$ \%\  Inhibition = 100\left[ 1\ \mathit{\hbox{--}}\ \left( Ab{s}_2\kern0.2em  sample\ \mathit{\hbox{--}}\ {A}_1\kern0.2em  sample\right)/\left( Ab{s}_2\kern0.2em  control\mathit{\hbox{--}}\  Ab{s}_1\kern0.2em  control\right)\right] $$

### Statistical analysis

Median inhibition concentrations (IC_50_) were obtained by plotting the graphic regression using Microcal™ Origin® software version 6.0 (Microcal Software, Inc., Northampton, USA) and presented as graphs using Excel for Windows (Microsoft, Inc., St. Louis, USA). Milliequivalence (mEq) values were obtained by division of IC_50_ of plant extracts by the specific IC_50_ standard used in each method.

## Results and discussion

Five plant extracts were tested using six different methods, namely radical scavenging with DPPH, ABTS, superoxide anion radicals, singlet oxygen scavenging, β-carotene bleaching and determination of total polyphenols to characterize antioxidant potential. Table 3 presents the free radical scavenger response of extracts in milliequivalents (mEq) of ascorbic acid for the radical scavenging tests and in mEq of gallic acid (mg of gallic acid corresponding to 1.0 g of dry extract) for determination of total polyphenols. The results of the calculations of 50 % inhibition concentrations (IC_50_) for extracts also are presented in Table 3.

**Table 3:** Total polyphenols and free radical scavenger activity (IC_50_ in μg/mL) of Amazonian medicinal plants

Species	Phenols	ABTS	DPPH	O_2_^-•^	^1^Δ_g_O_2_
*B. japurensis*	59.4	12.3 ± 0.2	8.4 ± 0.6	44.9 ± 6.4	1408 ± 113
*C. spruceanum*	60.2	5.0 ± 0.1	7.5 ± 0.9	18.2 ± 3.0	92.4 ± 24.8
*M guyanensis*	58.7	8.2 ± 0.3	28.4 ± 0.8	35.3 ± 3.0	517 ± 70.8
*P. nitida*	3.2	38.5 ± 1.9	49.9 ± 0.1	100 ± 9.9	In
*P. olacoides*	2.1	8.7 ± 0.3	29.7 ± 0.3	142 ± 16.5	715 ± 195
α-tocopherol	-	12.5 ± 0.6	10.4 ± 0.4	180 ± 14.4	nd
Ascorbic acid	-	4.8 ± 0.3	2.7 ± 0.3	32.9 ± 1.6	nd
Gallic acid	-	1.0 ± 0.1	1.1 ± 0.2	7.8 ± 1.2	nd
DABCO	-	nd	nd	nd	58.4 ± 6.0
TROLOX®	-	3.9 ± 0.1	5.6 ± 0.2	nd	nd

*Note*: *nd* not determined, *in.* inactive. Phenols are expressed in Equivalent of Gallic acid/g dry extract

The DPPH scavenging assay was chosen as a primary test to be performed in initial screening of extracts due to its relatively low cost and the high stability of this reagent. Mensor et al. [18] consider the DPPH method fast and easy for evaluation of the presence of antioxidant potential in biological samples with the great advantage that the test is prepared and executed at room temperature which eliminates the risk of thermal degradation of substances under study. *C. spruceanum* and *B. japurensis* extracts were the most active in the DPPH test exhibiting IC_50_ < 10 μg/mL (Table 3).

The ABTS radical scavenging test has been described exhaustively by many different authors and in general is useful for the evaluation of antioxidant activity of substances having lipophilic or hydrophilic properties, including flavonoids e carotenoids [14, 19, 20]. In this test, the most active extracts were those of *C. spruceanum*, *M. guyanensis* and *P. olacoides* which exhibited IC_50_ < 10 μg/mL (Table 3). IC_50_ values were lower than values for plant extracts considered antioxidant in the literature, such as *Calpurnia aurea,* an effective scavenger of the ABTS radical, with percentage inhibition 100 % [21]. Only samples exhibiting IC_50_ < 10 μg/mL are considered very active antioxidants as they have activity comparable to the antioxidant standards quercertin, β-carotene, ascorbic acid, gallic acid and Trolox® [22].

The ability of some extracts to scavenge free radicals in tests of antioxidant capacity, such as those based on DPPH and ABTS, does not mean that these extracts will perform readily where complex mechanisms are operating such as those in physiological substrates. For this reason, there is a need to verify the antioxidant effect in scavenging specific species such as superoxide anion radical (O_2_^•-^). O_2_^•-^ is produced constantly in organisms by diverse cellular processes, such as the electron transport chain in mitochondria, in microsomes and through enzymes like xanthine oxidase and NADPH oxidase and can be increased as part of certain pathologies [23]. Extracts of *C. spruceanum*, *M. guyanensis* and *B. japurensis* exhibited the highest superoxide anion radical scavenging activity as evidenced by IC_50_ of 18–45 μg/mL. It is noteworthy that these extracts are as active on a weight basis as several of the standards used, such as ascorbic acid [13].

Singlet oxygen (^1^Δ_g_O_2_) is the excited electronic state of molecular oxygen and is produced in general by photochemical reactions. It is reactive with a large number of biologically important molecules, including lipid membranes, through which peroxidation processes are initiated. Rubrene is a polycyclic hydrocarbon which is highly soluble in organic solvents and which auto-oxidizes in the presence of ambient light, generating ^1^ΔO_2_ from triplet oxygen (^3^Δ_g_O_2_), the ground state of molecular oxygen, present in air. For this reason, the assay involving rubrene is useful for the characterization of ^1^Δ_g_O_2_ scavenging action in the extracts tested [14]. The test has been used for the study of photoprotective and ^1^ΔO_2_ scavenging actions of substances isolated from fruit and other parts of plants, as well as fruit and vegetable juices [14, 22, 24]. The rubrene method did not provide good results in the present study. This may be due to the interference of the highly and diversely colored extracts at the relatively low visible light wave length used (440 nm). The lowest IC_50_ value was for *C. spruceanum* extract, which was comparable to that of the DABCO standard and lower than that for quercetin (Table 3).

**Fig. 1:**
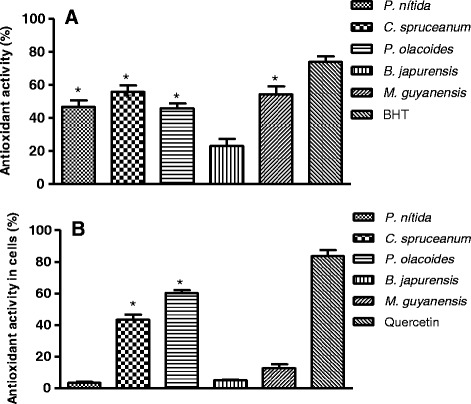
Antioxidant activity by β–carotene/linoleic bleaching assay (**a**) and cell-based assay (**b**) of ethanol extracts of five Amazonian medicinal plants at 100 μg/mL. Values are the mean ± S.D from three independent experiments. Significance was determined using Student’s-*t*-test (*p < 0.05 compared to control BHT (**a**) and quercetin (**b**)

When extracts were tested using the β–carotene/linoleic bleaching method *C. spruceanum, P. nitida* and *M. guyanensis* exhibited the best results which were evidenced by IC_50_ = 29.9 ± 4.5, 49.3 ± 3.7 and 53.3 ± 7.8, respectively while BHT exhibited IC_50_ = 21.9 ± 8.5 μg/mL. However, all tested extracts inhibited linoleic acid oxidation (Fig. 1).

In general, total polyphenols levels were related to antioxidant potential revealed in the other assays for the plant extracts under study. The plant extracts exhibiting the greatest antioxidant potential were those with the highest levels of total polyphenols, namely *B. japurensis*, *C. spruceanum* and *M. guyanensis*. This result is similar to that found by Cai et al. [19] who used the ABTS method to evaluate the antioxidant capacity of more than 112 medicinal plant species used in the treatment and prevention of cancer in chinese traditional medicine. Those results indicated a strong correlation between antioxidant activity and high levels of phenolic compounds.

**Fig. 2:**
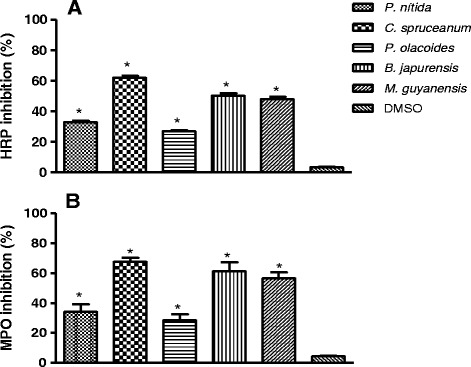
Inhibition of HRP (**a**) and MPO (**b**), using the amplex red and hydrogen peroxide of ethanol extracts of five Amazonian medicinal plants at 100 μg/mL. Values are the mean ± S.D from three independent experiments. Significance was determined using Student’s-*t*-test (*p < 0.05 compared to control DMSO)

Amplex Red® is a sensitive and specific probe for the detection of H_2_O_2_. It acts as substrate for endogenous peroxidases present in eosinophils and in neutrophils. It can be used not only in activated phagocytic cells but also in other types of cells or even in non-cellular systems [25]. Effective inhibition of HRP and MPO was demonstrated in Fig. 2. At a concentration of 100 μg/mL, the extracts of *P. nitida* and *P. olacoides* inhibited only 33 ± 1.21 % and 27 ± 1.18 %, respectively. However, *C. spruceanum*, *B. japurensis* and *M. guyanensis* exhibited significant activity (62 ± 2.26 %, 50 ± 2.59 % and 48 ± 2.16 % inhibition of HRP activity, respectively). Interestingly, we note similarities between the inhibitions of the peroxidase activities (HRP/MPO). In the MPO system, *P. nitida* and *P. olacoides* extracts inhibited activity by 34 ± 7.16 % and 28 ± 5.92 %, respectively. These values are lower than those observed for *C. spruceanum*, *M. guyanensis* and *B. japurensis* extracts which inhibited MPO by 67 ± 4.03 %, 61 ± 5.88 % and 56 ± 8.26 %, respectively.

MPO is a member of the heme peroxidase-cyclooxygenase superfamily and is abundantly expressed in neutrophils and to a lesser extent in monocytes and certain type of macrophages. MPO participates in an innate immune defense mechanism through formation of microbicidal reactive oxidants and diffusible radical species. A unique activity of MPO is its ability to use chloride as a co-substrate with hydrogen peroxide to generate chlorinating oxidants such as hypochlorous acid, a potent antimicrobial agent. However, evidence has emerged that MPO-derived oxidants contribute to tissue damage and the initiation and propagation of acute and chronic vascular inflammatory disease. The fact that circulating levels of MPO have been shown to predict risks for major adverse cardiac events and that levels of MPO-derived chlorinated compounds are specific biomarkers for disease progression, has attracted considerable interest in the development of therapeutically useful MPO inhibitors [26]. Thus, the anti-inflammatory activities of the medicinal plants evaluated in this study may be related to inhibition of the enzyme MPO demonstrated herein. This inhibition can occur in several ways: (i) due to presence of oxidizable constituents capable of acting on the prosthetic group causing enzyme inhibition, (ii) not directly by enzyme inhibition, but by inhibition of oxidable species generated and (iii) by chelation of metals such iron or copper which are necessary for enzyme activity [26].

## Conclusions

In conclusion, low IC_50_ values demonstrated the excellent free radical scavenging and antioxidant potential of *B. japurensis*, *C. spruceanum* and *M. guyanensis*. It is important to emphasize that the data presented in this study are not yet available for any of these species and reveal the great potential of the Amazon medicinal flora as a source of new bioactive, antioxidant extracts with potential therapeutic uses.
