# Erratum to: Antioxidant activity and peroxidase inhibition of Amazonian plants extracts traditionally used as anti-inflammatory

**DOI:** 10.1186/s12906-016-1097-x

**Published:** 2016-04-14

**Authors:** Fabiano de S. Vargas, Patricia D. O. Almeida, Ana Paula A. de Boleti, Maria M. Pereira, Tatiane P. de Souza, Marne C. de Vasconcellos, Cecilia Veronica Nunez, Adrian M. Pohlit, Emerson S. Lima

**Affiliations:** Faculdade de Ciências Farmacêuticas, Universidade Federal do Amazonas - UFAM, Rua Alexandre Amorim, 330, Bairro Aparecida, Cep: 69010-300 Manaus, Amazonas Brazil; Coordenação de Tecnologia e Inovação-CoTI, Instituto Nacional de Pesquisas da Amazônia-INPA, Avenida André Araújo, Petrópolis, CEP: 69067375 Manaus, Amazonas Brazil

Following the publication of the original article [1] it was brought to our attention that one of the authors’ names was incorrectly displayed on the website and PDF version of the article. At current, the author name appears as ‘Fabiano S. de Vargas’, whereas the name should appear correctly as Fabiano de S. Vargas. We apologise for any confusion caused.
